# Molecular mechanism and potential role of mitophagy in acute pancreatitis

**DOI:** 10.1186/s10020-024-00903-x

**Published:** 2024-09-03

**Authors:** Lili Zhu, Yunfei Xu, Jian Lei

**Affiliations:** 1 Department of Pathology, The Affiliated Cancer Hospital of Xiangya School of Medicine, Central South University, Hunan Cancer Hospital, Changsha, China; 2 Department of Pathophysiology, School of Basic Medical Sciences, Central South University, Changsha, 410078 Hunan China; 3 Postdoctoral Research Station of Biology, School of Basic Medical Science, Central South University, Changsha, Hunan China

**Keywords:** Pancreatitis, Mitochondrial dysfunction, Mitophagy, Mitophagosome, Lysosome

## Abstract

Acute pancreatitis (AP) is a multifaceted inflammatory disorder stemming from the aberrant activation of trypsin within the pancreas. Despite the contribution of various factors to the pathogenesis of AP, such as trypsin activation, dysregulated increases in cytosolic Ca^2+^ levels, inflammatory cascade activation, and mitochondrial dysfunction, the precise molecular mechanisms underlying the disease are still not fully understood. Mitophagy, a cellular process that preserves mitochondrial homeostasis under stress, has emerged as a pivotal player in the context of AP. Research suggests that augmenting mitophagy can mitigate pancreatic injury by clearing away malfunctioning mitochondria. Elucidating the role of mitophagy in AP may pave the way for novel therapeutic strategies. This review article aims to synthesize the current research findings on mitophagy in AP and underscore its significance in the clinical management of the disorder.

## Introduction

Acute pancreatitis (AP) is an inflammatory disorder of the pancreas, frequently precipitated by factors such as gallstones and alcohol misuse (Gardner 2021). The disease arises from the uncontrolled activation of prematurely activated trypsin, which leads to injury of pancreatic acinar cells, resulting in pancreatic edema, hemorrhage, and potentially necrosis. In its severe manifestations, AP can evolve into systemic inflammation and multiorgan dysfunction syndrome (Wen et al. 2022; Jung et al. 2011; Pandol et al. 2007; Vege et al. 2018; Garg and Singh 2019). AP can be categorized into two principal forms based on disease severity: acute necrotizing pancreatitis and interstitial edematous pancreatitis. Necrotizing pancreatitis, although less prevalent, accounts for the majority of AP-related mortality (Banks et al. 2013). The incidence of AP is increasing globally, exerting a substantial burden on hospital admissions for gastrointestinal disorders (Manizhashvili and Lomidze 2020; Lee and Papachristou 2019; Goodman et al. 2020). Current statistics indicate an incidence rate of 33.74 cases per 100,000 person-years and a mortality rate of 1.60 deaths per 100,000 person-years for AP, with a continuing upward trend (Xiao et al. 2016; Habtezion et al. 2019). Despite advancements in elucidating the pathogenic mechanisms of AP, which include intrapancreatic trypsinogen activation (Saluja et al. 2019; Mayerle et al. 2019), endoplasmic reticulum (ER) stress (Mao et al. 2022; Biczo et al. 2018), cytokine release (Liu et al. 2017; Wang et al. 2017; Bhatia 2005), dysregulation of calcium homeostasis (Criddle 2016; Pallagi et al. 2022; Du et al. 2022), defective autophagy (Piplani et al. 2019; Gukovsky et al. 2012; Ji et al. 2016, 2022; Lv et al. 2022; Wang et al. 2021), mitochondrial dysfunction (Biczo et al. 2018; Ghosh et al. 2018; Yuan et al. 2021), and disturbances in ATP synthesis (Mukherjee et al. 2016; Park et al. 2020), significant gaps in knowledge remain. Additional research is imperative to comprehensively understand the pathogenic processes and guide therapeutic strategies’ development. AP is a potentially lethal condition characterized by high morbidity and mortality rates, largely due to its intricate pathophysiology (Pandol et al. 2007; Peery et al. 2019). The primary treatment for AP consists of fluid resuscitation and supportive measures, as no current therapeutic agents can entirely alter the disease course (Vege et al. 2018; Zheng et al. 2021). In response, ongoing research endeavors are focused on enhancing the fundamental scientific understanding of AP, to identify novel targets for pharmacological and genetic interventions.

Autophagy is a cellular process that facilitates the degradation of large molecules or damaged organelles within cells. It can be categorized into non-selective forms, such as microautophagy and macroautophagy (Dikic and Elazar 2018), or selective forms, including mitophagy, pexophagy, ER-phagy, and ribosomal autophagy (Liu et al. 2020; Green and Levine 2014). Among these, mitophagy is a critical type of selective autophagy that is essential for maintaining cellular and mitochondrial homeostasis. Mitochondria are indispensable for energy metabolism and cellular physiology; thus, damaged or dysfunctional mitochondria can precipitate a variety of pathologies. External stimuli, including reactive oxygen species (ROS), ischemia, nutrient deprivation, and cellular senescence, can induce mitochondrial damage and depolarization. Mitophagy selectively engulfs these impaired mitochondria into autophagosomes, fusing with lysosomes for degradation, thereby preserving intracellular stability and cellular homeostasis. Mitophagy is vital for mitigating inflammation triggered by ROS and danger-associated molecular patterns (DAMPs), clearing dysfunctional and damaged mitochondria in eukaryotic cells, and promoting mitochondrial quality control. It is crucial for sustaining mitochondrial function and preventing the accumulation of defective organelles that could lead to cellular and tissue damage (Palikaras et al. 2018). Mitophagy plays a pivotal role in inflammatory diseases, including acute lung injury (Luo et al. 2019; Jing et al. 2020; Mannam et al. 2016; Leermakers et al. 2020), sepsis (Zhu et al. 2018, 2021; Mannam et al. 2014), cardiovascular diseases (Marek-Iannucci et al. 2021; Wang et al. 2022a, b; Quiles and Gustafsson 2022), hepatic injury (Mao et al. 2022; Zhong et al. 2022; Lu et al. 2021), renal injury (Yu et al. 2021; Su et al. 2023; Duan et al. 2022), and various conditions caused by inflammasome activation (Yuk et al. 2020; Zhang et al. 2019; Ojeda et al. 2018; Li et al. 2019; Liu et al. 2018; Singh et al. 2017; Wu et al. 2016). Consequently, mitophagy is a fundamental mechanism with significant implications in human disease pathophysiology.

Empirical research has demonstrated that the selective targeting of damaged mitochondria and the restoration of mitochondrial function are pivotal strategies in the treatment of AP. Mitophagy has emerged as a promising mechanism in this therapeutic context, with recent studies elucidating the underlying pathways and molecular mechanisms involved (Biczo et al. 2018; Pallagi et al. 2022; Piplani et al. 2019; Ku et al. 2020; Choi and Kim 2020; Swain et al. 2020; Tóth et al. 2019; Armstrong et al. 2019; Ampawong et al. 2017). This review article aims to synthesize the latest findings in mitophagy research, underscoring its significance in the management of AP. Furthermore, we will explore potential therapeutic targets associated with mitophagy that may be leveraged to combat this debilitating condition.

### Cell death and AP

Distinct forms of cell death, such as apoptosis, pyroptosis, necroptosis, and autophagy, are activated during AP and contribute to its progression (Sendler et al. 2016a, b). These various forms of cell death are intricately interconnected, underscoring the necessity of comprehending their interrelationships and functions within the pathology of AP and in the pursuit of potential therapeutic targets.

In AP, multiple forms of cell death have been observed. The premature activation of intracellular proteases in acinar cells triggers necrotic cell death, which is an unregulated response to damage. In AP animal models, a certain percentage of acinar cells undergo apoptosis, and this rate is inversely correlated with the severity of AP (Gukovskaya and Gukovsky 2011). Autophagy, a complex and lysosome-mediated process for degrading cytoplasmic organelles and long-lived proteins, when impaired, as indicated by vacuole accumulation, is associated with AP (Choi and Kim 2020; Mareninova et al. 2009; Debnath et al. 2023). Notably, there is a debate regarding the role of impaired autophagy in inducing cell death through the accumulation of damaged mitochondria, leading to an inflammatory response via a ROS-dependent mechanism (Mareninova et al. 2015; Iwahashi et al. 2018; Zhou et al. 2022), or whether autophagy prevents an inflammatory response (Hashimoto et al. 2008; Gukovsky and Gukovskaya 2015; Larabi et al. 2020). Necroptosis, triggered by factors released in the early phase of AP in response to protease activation, involves the formation of the necrosome involving receptor-interacting serine/threonine-protein kinase 1 (RIPK1) or RIPK3, resulting in the phosphorylation of mixed lineage kinase domain-like (MLKL), ultimately resulting in membrane rupture (He et al. 2016). Interventions targeting necroptosis can ameliorate the severity of AP (He et al. 2009; Louhimo et al. 2016). Additionally, necroptosis can also lead to the release of damage-associated molecular patterns (DAMPs) and activate the NOD-like receptor pyrin domain-containing protein 3 (NLRP3) pathway, resulting in another form of cell death known as pyroptosis (Malik and Kanneganti 2017). Inhibitors of pyroptosis such as lactate, beta-hydroxybutyrate, and aspartate have demonstrated potential in AP. The term “pyroptosis” refers to the activation of the inflammasome through NLRP3. The activation of NLRP3 involves the cleavage of pro-IL-18 and IL-1β, as well as the release of high-mobility group box 1 (HMGB1), which requires lysosomal rupture, calcium influx, and ROS production from mitochondria, making it closely linked to the development of AP (Hoque et al. 2011, 2014).

Cell death forms in AP involve the activation of caspase 3, resulting in apoptosis and pyroptosis (Vince and Silke 2016; Jiang et al. 2020). Necroptosis can also transition to pyroptosis via caspase 8 activation, thus adding complexity to the cell death switch (Fritsch et al. 2019). The reasons why certain patients progress to a necrotizing form of AP remain unclear, yet they might be associated with a shift toward pyroptosis and necroptosis. Inhibiting pyroptosis, for instance by using Ringer’s lactate for resuscitation, or necroptosis with necrostatin, could potentially serve as treatment approaches for AP (Al Mamun et al. 2022; Ouyang et al. 2021).

The role of premature protease activation vis-à-vis uncontrolled inflammation in inducing cell death in AP remains equivocal, although trypsin is known to play a crucial role. One study demonstrated that active trypsin directly affects lysosomal stability, resulting in lysosomal rupture and the release of cathepsins into the cytosol, thereby inducing apoptosis or necrosis in a dose-dependent manner (Talukdar et al. 2016). However, another study found that inhibiting protease activation, especially trypsin, reduced the rate of apoptosis but not necrosis (Sendler et al. 2016a, b). These observations were made in isolated acinar cells mimicking the early stages of pancreatitis. Inflammation is both the source and consequence of cell death in pancreatitis, as evidenced by the involvement of pyroptosis and necroptosis.

### The characteristics and regulations of mitophagy

**Fig. 1:**
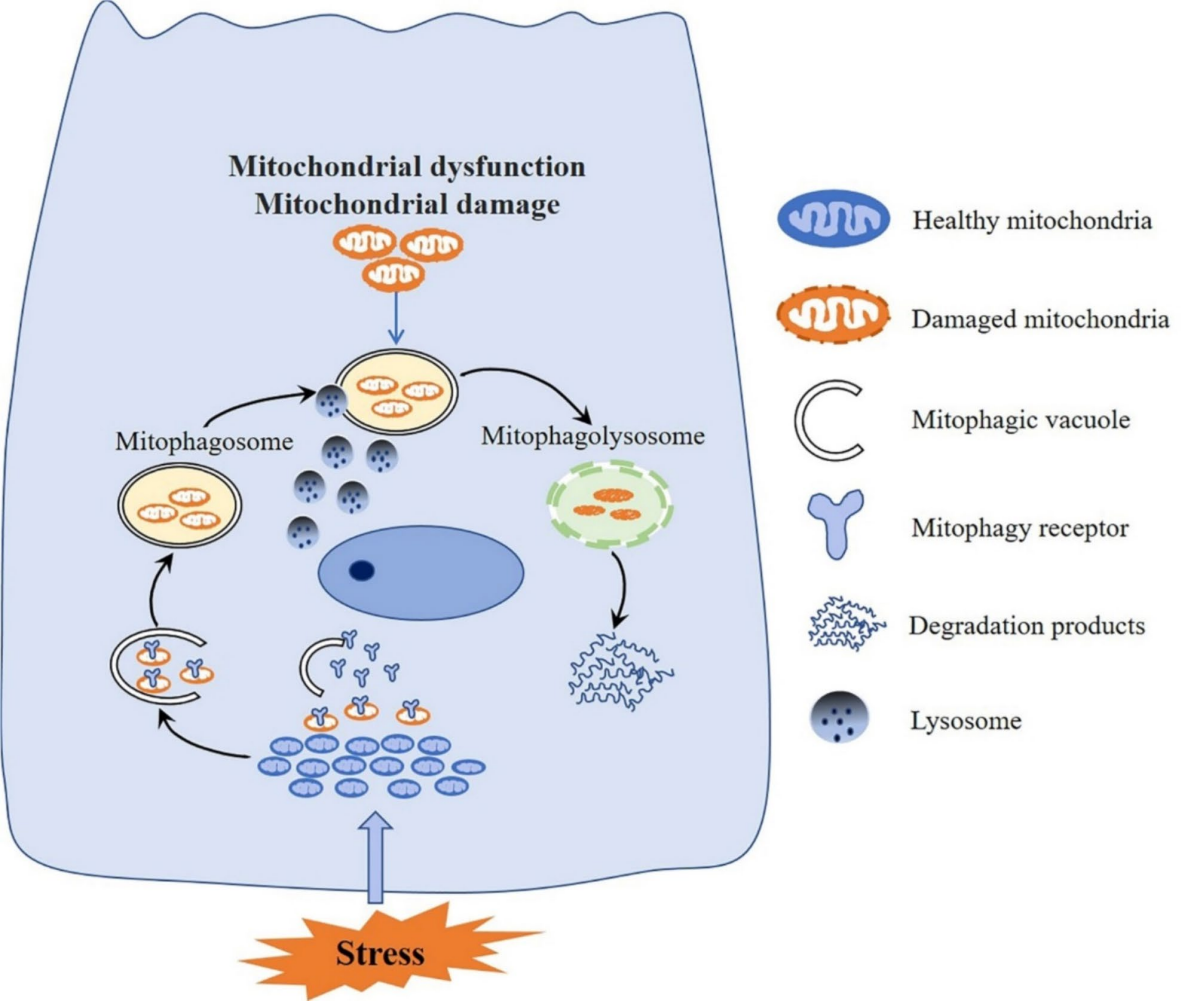
The process of mitophagy

**Fig. 2:**
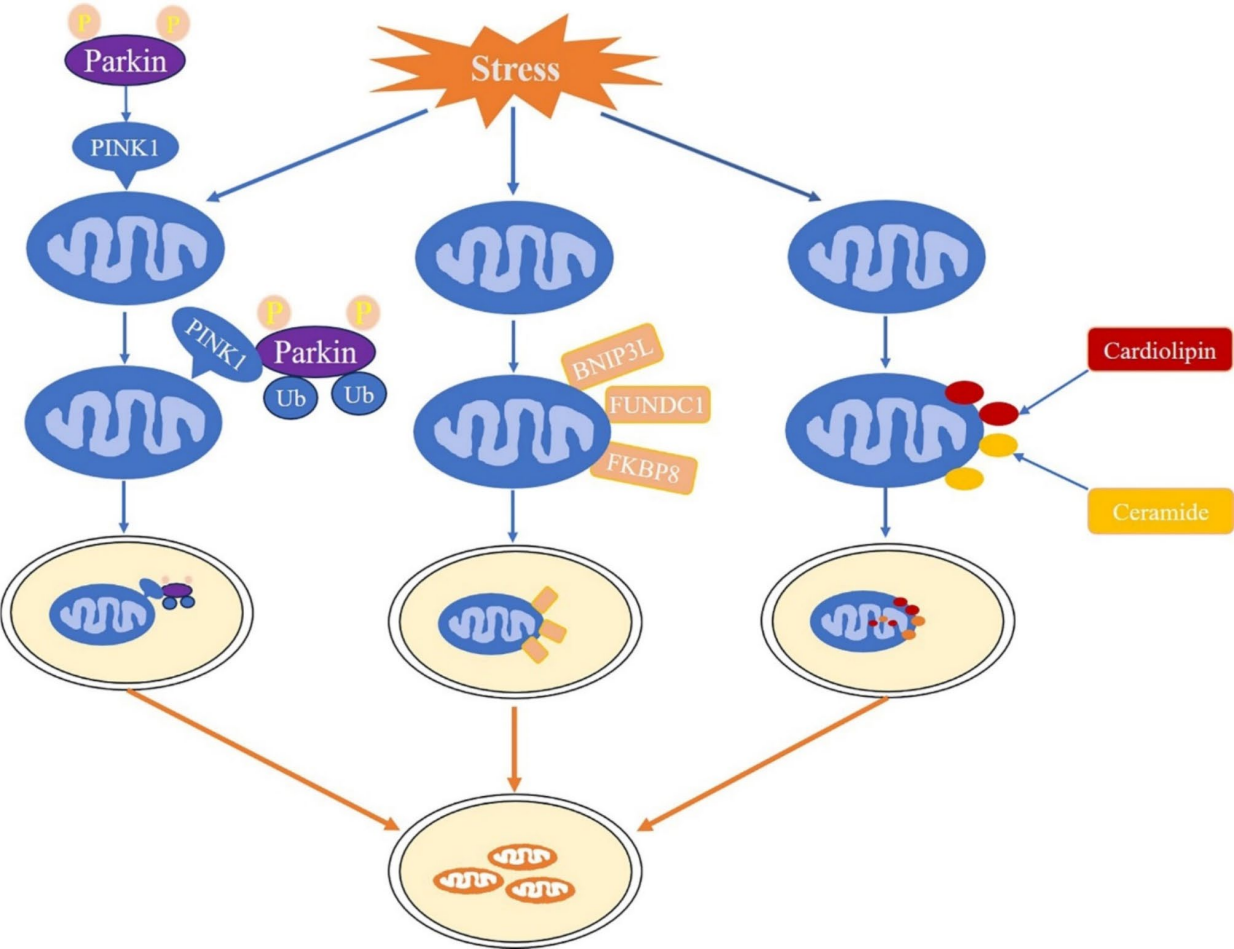
The molecular mechanism of mitophagy

Mitophagy is a dynamic cellular process that selectively eliminates dysfunctional or damaged mitochondria to maintain normal cellular function. It can be triggered by various stimuli, with depolarization being the most widely studied. Other triggers include hypoxia and NAD+. Mitophagy occurs in diverse physiological contexts, for instance during the differentiation of red blood cells and retinal ganglion cells, where it is distinct and non-detrimental. The process of mitophagy involves the formation of double-membrane autophagosomes that enwrap damaged mitochondria and cytoplasmic components. These autophagosomes subsequently fuse with lysosomes to form autophagy-lysosome structures, within which the contents are degraded by lysosomal enzymes. This process is depicted in Fig. 1. Overall, mitophagy contributes to maintaining cellular homeostasis and organelle renewal (Hirano and Manabe 1993). It functions through three main mechanisms: ubiquitin-mediated mitophagy, including phosphatase and tensin homolog-induced putative kinase protein 1 (PINK1)-Parkin (PARK)-dependent mitophagy, outer mitochondrial membrane (OMM) receptor-mediated mitophagy, and lipid-mediated mitophagy. We summarize its mechanism in Fig. 2.

Under stress, damaged mitochondria and cytoplasmic components are encapsulated within a double-membrane autophagic vacuole. Subsequently, the outer membrane of the autophagosome fuses with lysosomes, giving rise to an autophagy-lysosome complex within which the contents of the autophagosome, along with its inner membrane, are degraded by lysosomal hydrolases.

### PINK1-PARK-mediated mitophagy

The PINK1-PARK pathway is the most extensively studied mechanism of mitophagy. PINK1 is a mitochondrial protein kinase encoded by the PARK6 gene, and PARK is a cytosolic E3 ubiquitin ligase encoded by the PARK2 gene. Ordinarily, PINK1 is localized to the mitochondria and is translocated to the mitochondrial inner membrane (MIM), where it is cleaved, inactivated, and degraded. However, when mitochondria depolarize, PINK1 fails to translocate to the MIM and accumulates at the outer mitochondrial membrane (OMM). This accumulation triggers the activation of PARK recruitment and phosphorylation, which is inactive in healthy mitochondria and is located in the cytoplasm (Hirano and Manabe 1993; Hara et al. 1994). Phosphorylated PARK promotes the ubiquitination of mitochondrial membrane proteins and the recruitment of autophagy receptors such as optineurin, p62, nuclear dot protein 52, and breast cancer susceptibility 1 to mitochondria. This process drives the damaged mitochondria towards the autophagy pathway, engulfing them by growing phagophores or isolation membranes. Eventually, mitophagy aids in removing damaged mitochondria and maintains cellular homeostasis.

### OMM receptor-mediated mitophagy

OMM receptor-mediated mitophagy is another crucial mechanism for the selective removal of damaged mitochondria. These receptors are typically mitochondrial proteins embedded in the OMM via a transmembrane domain. They possess an LC3-interacting region (LIR) motif that facilitates the recruitment of LC3 and the growing mitophagophore to the targeted mitochondria (Petersen et al. 2021). In mammals, several OMM receptors have been identified, such as NIP3-like protein X (NIX or BNIP3L), FUN14 domain-containing 1 (FUNDC1), and FK506 Binding Protein 8 (FKBP8). BNIP3 is usually present in the cytosol as an inactive monomer. Hypoxia induces the upregulation of BNIP3, causing it to be anchored to the OMM via its C-terminal transmembrane (TM) domain while exposing the N-terminal domain to the cytosol (Greer et al. 2015). There is an LIR motif at the N-terminal region of BNIP3, which responds to the interaction with LC3. Phosphorylation and dimerization are reported to enhance the activity of NIX, which is transcriptionally regulated by hypoxia-inducible factor-1 alpha (HIF-1α) stabilization during hypoxia-induced mitophagy (Wang et al. 2022a, b). BNIP3, an OMM receptor, is typically found in its inactive monomeric form in the cytosol. However, during hypoxia, BNIP3 is upregulated and attaches to the OMM through its C-terminal TM domain, exposing its N-terminal domain to the cytosol. The N-terminal region of BNIP3 contains the LIR motif, which interacts with LC3 to initiate mitophagy. Phosphorylation and dimerization have been found to boost the activity of NIX, another OMM receptor regulated by HIF-1α during hypoxia-induced mitophagy. BNIP3 is involved in PINK1-PARK mitophagy by stabilizing PINK1 on the OMM, facilitating dynamin-related protein 1 (DRP1) translocation, and preventing the interaction between beclin1 and B-lymphoma-2 (Antonucci et al. 2015; Chen et al. 2020; Mareninova et al. 2022). FUNDC1, an integral OMM protein, mediates hypoxia-induced mitophagy by interacting with LC3 through its LIR motif and three transmembrane (TM) domains (Lee et al. 2021). Hypoxia promotes the phosphorylation of FUNDC1, thereby activating its interaction with LC3 and augmenting mitophagy. FKBP8, another MOM protein, has an LIR motif at its N-terminus. It recruits autophagosomes to mitochondria for mitophagy by interacting with LC3A. In summary, OMM receptor-mediated mitophagy is a crucial regulatory mechanism in mitophagy.

### Lipid-mediated mitophagy

Following mitochondrial stress, specific lipids relocate to the OMM and directly interact with LC3, facilitating the recruitment of autophagosomes to mitochondria to initiate mitophagy. Cardiolipin, a mitochondrial membrane phospholipid, functions as a mitophagy receptor by translocating from the mitochondrial inner membrane (MIM) to the OMM following mitochondrial injuries (Armstrong et al. 2018). At the OMM, cardiolipin directly recruits LC3 to mitochondria by binding to its N-terminal helix to induce mitophagy (Du et al. 2017). Ceramide, a bioactive sphingolipid, consists of a sphingosine backbone and a fatty acyl chain. The type of ceramide is determined by the number of carbon atoms in the fatty acyl chain. Its de novo synthesis is controlled by fatty-acyl CoA and six different ceramide synthases (CerS), including CerS1 to CerS6. CerS1 and its metabolic product C18-ceramide have been demonstrated to selectively induce lethal mitophagy. C18-ceramide induces the upregulation of LC3B-II and co-localizes with LC3B-II at mitochondria, thereby leading to the recruitment of autophagosomes to mitochondria and mediating mitophagy.

Mitophagy is carried out through three distinct pathways. The PINK1-PARK-mediated mitophagy pathway: Stress-induced mitochondrial depolarization results in the accumulation of PINK1 in the OMM, thereby activating the recruitment and phosphorylation of PARK. This, in turn, leads to the ubiquitination of mitochondrial membrane proteins and the recruitment of autophagy receptors, ultimately propelling mitochondria into the autophagy pathway. The OMM receptor-mediated mitophagy pathway: OMM receptors like BNIP3L, FUNDC1, and FKBP8 play a crucial role in this pathway. Hypoxia triggers the activation of OMM receptors, exposing their N-terminal domain, which interacts with LC3 through the LIR motif and is recruited to mitochondria, mediating mitophagy. The lipid-mediated mitophagy pathway: Following mitochondrial stress, cardiolipin, and ceramide are transferred from the mitochondrial inner membrane (MIM) to the OMM, recruiting LC3 to the mitochondria and inducing mitophagy.

### The potential role of mitophagy in pancreatitis

The onset of AP primarily involves pancreatic acinar cells, which are responsible for the synthesis, transportation, storage, and secretion of digestive enzymes. The normal functioning of acinar organelles, such as the endoplasmic reticulum, mitochondria, and endolysosomal-autophagy system, is of crucial significance for maintaining their physiological functions. In recent years, the dysfunction of mitochondrial function and mitophagy has emerged as a significant mechanism in the development of AP. Earlier studies conducted in 1988 by K.T. Lee and P. Ching Sheen revealed alterations in the ultrastructure of mitochondria in pancreatic acinar cells of AP patients (Lee and Ching Sheen 1988). Subsequent reports have documented mitochondrial swelling, fractured mitochondrial cristae, mitochondrial fragility, and mitochondrial dysfunction in AP (Hirano and Manabe 1993; Hara et al. 1994; Monney-Faller 1979; Parisi de Fabro et al. 1990). The timely degradation and clearance of damaged mitochondria are critical in determining the progression of AP. In other words, mitophagy is a key player in the development of AP. Notably, the susceptibility of different etiologies to mitophagy defects may vary depending on the underlying mechanisms. Alcohol exposure can impair mitochondrial function and autophagy, resulting in the accumulation of damaged mitochondria (Farooq et al. 2021). This could make alcohol-induced AP particularly prone to mitophagy defects. Genetic mutations leading to hereditary AP can affect the function of pancreatic enzymes or the structure of the pancreatic ducts (Liu et al. 2014; Verny et al. 2008). Although these mutations may not directly impact mitophagy, the recurrent nature of hereditary AP could lead to cumulative mitochondrial damage and impaired mitophagy over time. The obstruction of the pancreatic duct by gallstones may cause ischemia and reperfusion injury, which can cause mitochondrial damage (Lee and Ching Sheen 1988). However, the direct effect on mitophagy might not be as prominent as in alcohol-induced AP. Understanding the relationship between the etiology of AP and mitophagy defects is crucial for devising targeted therapies. For example, therapies that enhance mitophagy could be particularly advantageous in alcohol-induced AP, as mitochondrial damage is a crucial factor. Conversely, addressing the underlying genetic mutations in hereditary AP could aid in preventing the progression of mitochondrial damage and improving outcomes. More research is required to explore these relationships and to develop specific interventions for the different etiologies of AP.

### Mitochondrial dysfunction during AP

Mitochondrial function is of crucial significance for normal pancreatic protein synthesis and sorting, as well as for the maintenance of intracellular organelles and the secretion of enzymes. However, AP-induced mitochondrial dysfunction has been widely reported in numerous studies, resulting in pancreatic endoplasmic reticulum stress, impaired autophagy, and dysregulation of lipid metabolism (Biczo et al. 2018).

AP-induced calcium overload in mitochondria significantly contributes to mitochondrial dysfunction. Stimulation of Piezo1 or Orai1 triggers the opening of transient receptor potential vanilloid subfamily 4 (TRPV4) channels, resulting in sustained intracellular calcium elevation and subsequent intracellular organelle dysfunction (Swain et al. 2020). Prolonged or excessive calcium influx leads to mitochondrial permeability transition pore (MPTP) permeabilization, generation of reactive oxygen species, decreased mitochondrial ATP production, and ultimately, mitochondrial dysfunction, cell death, and exacerbation of AP (Petersen et al. 2021). The MPTP channel plays a vital role in regulating calcium ion homeostasis in AP. Inhibition of the MPTP, either genetically or pharmacologically, has been shown to enhance the outcome of AP in animal models. Studies have demonstrated that genetic and pharmacological inhibition of the MPTP protects mitochondrial homeostasis and cell function in pancreatic ductal cells affected by AP-inducing factors (Tóth et al. 2019). Notably, the mechanisms of MPTP opening in experimental pancreatitis are specific to the models used in Table 1. In cerulein-induced AP, mitochondrial calcium overload leads to MPTP opening (Mukherjee et al. 2016). Conversely, in arginine-induced pancreatitis, MPTP opening is mediated by inhibition of ATP synthase (Biczo et al. 2018), and in alcohol-induced pancreatitis, it is mediated by a decrease in the ratio of nicotinamide adenine dinucleotide to nicotinamide adenine dinucleotide plus hydrogen due to oxidative alcohol metabolism (Shalbueva et al. 2013). However, regardless of the underlying mechanisms, MPTP opening in all models of pancreatitis is dependent on cyclophilin D (CypD). The activation of CypD in acinar cells by pancreatitis-inducing factors causes the opening of the MPTP channel, resulting in calcium overload and subsequent cell death.

The biosynthesis and dynamics of mitochondrial homeostasis are disrupted in AP. Studies have demonstrated that shortly after the induction of AP, there is a significant reduction in mitochondrial oxygen consumption and ATP production, resulting in a decreased ATP/O ratio. These mitochondrial dysfunctions are accompanied by alterations in mitochondrial dynamics, as evidenced by differential expression of optic atrophy 1 (OPA-1) and DRP-1, along with modifications in mitochondrial fission, elongation, and mitophagy during the acute phase of experimental mild pancreatitis in rats. Severe AP caused by alcohol abuse is mainly attributed to the accumulation of non-oxidized ethanol metabolites, such as fatty acid ethyl esters, in the pancreas. Pancreatic acinar cells exposed to a combination of ethanol and palmitoleic acid (EtOH/POA) exhibit increased levels of mitochondrial dysfunction, characterized by decreased mitochondrial membrane polarization (Ku et al. 2020). Cigarette toxins, in the context of smoking and AP, can mediate both pro-inflammatory and anti-inflammatory pathways, leading to transcriptional changes in pancreatic acinar cell function, thiamine deficiency, and mitochondrial dysfunction, thereby increasing the propensity for AP occurrence. These findings suggest that inflammation may also play a role in AP-induced mitochondrial dysfunction (Greer et al. 2015).

**Table 1:** The mechanisms of MPTP opening in AP

Model	Key mechanisms	References
Cerulein	Mitochondrial Ca^2+^ overload	Mukherjee R, Mareninova OA, Odinokova IV, et al., 2016
Arginine	ATP synthase inhibition	Biczo G, Vegh ET, Shalbueva N, et al., 2018
Alcohol	Decreased NAD/NADPH	Shalbueva N, Mareninova OA, Gerloff A, et al., 2013

### Mitophagy pathways in AP

Mitophagy, a selective autophagy process, is triggered in response to mitochondrial dysfunction induced by AP. This process serves to clear the accumulated damaged mitochondria. Moreover, zymophagy, another selective autophagy pathway, is also initiated early in AP. The activation of these selective autophagy pathways might account for the mild, self-limiting, and more frequently observed clinical manifestation of AP.

In caerulein (CAE)-induced AP, HPDE6-C7 cells exhibited elevated levels of mitochondrial Ca²⁺, mitochondrial membrane potential depolarization, mitophagosome formation, and enhanced protein expression of mitophagy markers like the ratio of LC3II/I, PINK1, and PARK, while showing decreased protein expression of p62 and translocase of the outer mitochondrial membrane complex subunit 20 (TOMM20) (Lei et al. 2024). Transmission electron microscopy of the pancreas revealed abnormalities in mitochondrial structure, such as disrupted cristae, a clearer matrix, and autolysosomes with remaining mitochondrial structures after 60 min of CAE administration. However, mitochondrial morphology returned to normal 48 h after the first CAE injection, suggesting the occurrence of mitophagy in the early stages of mild experimental pancreatitis (Vanasco et al. 2021). Recent studies have demonstrated that mitophagy is regulated by the PINK1/PARK2-mediated pathway and that LC3B and TOMM20 colocalize in pancreatic acinar cells during experimental AP. Moreover, the loss of PINK1 did not influence basal mitophagy in all tissues except pancreatic islets, although it disrupted depolarization-induced PARK activation (McWilliams et al. 2018). Mitophagy regulated by the PINK1/PARK2 pathway has been demonstrated to alleviate pancreatitis by controlling NLRP3-related inflammation pathways, thus highlighting the crucial role of mitochondria in the integration of autophagy and inflammation (Zhang et al. 2021). During AP, PARK1 recognizes and recruits damaged mitochondria for autophagy degradation. Vanasco et al. analyzed mitochondrial dynamics and function during selective autophagy in pancreatic acinar cells during mild experimental AP in rats and cell models, by using the pMITO-RFP-GFP plasmid to label the autophagic degradation of mitochondria and assessing the expression and redistribution of the ubiquitin ligase PARK1. They reported that vacuole membrane protein-1 (VMP1) plays a critical role in the mitophagy process during AP, suggesting a novel DRP1-PARK1-VMP1 selective autophagy pathway that mediates the selective degradation of damaged mitochondria by mitophagy in AP. Moreover, further research has shown that Trim33, a key E3 ligase enzyme that mediates trypsin ubiquitination, upregulates VMP1 expression and exerts a protective role in AP (Wang et al. 2022a, b).

In AP, cytokines are key mediators of the inflammatory response that can either protect against or contribute to tissue damage (Yu et al. 2015). Mitophagy plays a crucial role in regulating inflammation by controlling the release of inflammatory cytokines. AP induces the compromise of mitochondrial DNA and other mitochondrial constituents, resulting in their release into the cytosol. These released elements act as DAMPs (Zhang et al. 2010), engaging the innate immune system through receptors such as Toll-like receptor 9 or the NLRP3 inflammasome (Lu et al. 2023). By clearing damaged mitochondria, mitophagy can prevent the release of DAMPs. By averting the accumulation of damaged mitochondria, mitophagy may reduce apoptosis-related inflammation and subsequent cytokine production (Scaini et al. 2022). Additionally, efficient mitophagy helps maintain the redox balance within cells to limit cytokine production (Bharath et al. 2020). The formation of neutrophil extracellular traps (NETs) in AP is a crucial aspect of the immune response and is closely associated with mitophagy. NETs can augment local inflammation and oxidative stress, which are known to impair mitochondrial function. This impairment can result in an accumulation of damaged mitochondria, thereby necessitating an increase in mitophagy to eliminate these damaged organelles (He et al. 2023). The components of NETs, such as DNA and histones, have the potential to interfere with mitochondrial dynamics and function, leading to mitochondrial damage (Skoglund et al. 2021). This damage serves as a signal for the need for mitophagy to clear the damaged mitochondria. NET formation can activate immune cells to engulf and digest NETs. And their activation might potentially modulate the mitophagic process in response to NETs. NETs might influence the autophagic flux, which encompasses the formation, maturation, and degradation of autophagosomes (Maugeri et al. 2014). Disruption of this flux could hamper the removal of damaged mitochondria via mitophagy. In short, mitophagy can contribute to either the mitigation or exacerbation of the inflammatory response observed in AP, contingent upon its efficiency and regulation.

Mitophagy can activate a vast array of downstream signaling cascades, thereby initiating a diverse range of pathological responses (Wang et al. 2023; Youle and Narendra 2011). When mitochondrial function is compromised, cytochrome C is translocated from the mitochondrial intermembrane space into the cytosolic compartment (Morales-Cruz et al. 2014). This pivotal event triggers the activation of caspase-9, initiating a sequential caspase cascade that ultimately leads to apoptosis (Würstle et al. 2012). Additionally, mitochondrial abnormalities can enhance the production of ROS, which not only damages cellular components but also activates signaling pathways integral to stress responses, including the p53 pathway (Lee et al. 2022). ROS can also activate kinases like JNK and MAPK, which are crucial in determining cell fate (Lei et al. 2018). Mitochondrial ROS and cytosolic mitochondrial DNA can trigger the NF-κB signaling pathway, a pivotal player in orchestrating inflammation, immune responses, cell survival, and stress reactions (Ye et al. 2023; Harding et al. 2023). Sirtuins (SIRT), a prominent family of proteins, act as crucial regulators in cellular processes in the face of stress, such as calorie restriction and DNA damage. The impairment of mitochondrial function can significantly impact the activity of SIRT, including SIRT1 and SIRT3, which are instrumental in modulating mitochondrial function and stress resilience (Tabassum et al. 2023). The activation of these pathways has profound effects on cellular functionality and viability. A comprehensive understanding of these pathways is crucial for the development of therapeutic strategies aimed at counteracting the consequences of mitochondrial dysfunction and defective mitophagy in AP.

### Impaired autophagy and mitophagy exacerbate AP

Basal autophagy plays a crucial role in maintaining pancreatic acinar cell homeostasis and protein synthesis, thereby preventing ER stress (Antonucci et al. 2015). However, during AP, premature activation of trypsinogen in pancreatic acinar cells triggers autophagy and mitophagy, resulting in the accumulation of dysfunctional organelles, such as mitochondria. Autophagic vacuoles accumulate, impairing lysosomal degradation and ultimately leading to acinar cell death. There is evidence to suggest that mitophagy defects might indeed be present in AP. Studies have demonstrated that in the context of pancreatitis, there is an increase in mitochondrial damage and a disruption in the normal processes of mitophagy (Vanasco et al. 2021; Zhang et al. 2021). This suggests that mitophagy defects could be a common feature in the disease.

It has been proposed that autophagy is impaired in AP. By employing ATG7Δpan mice, in which the essential ATG7 is lacking in pancreatic epithelial cells, Antonucci et al. discovered that ATG7 loss results in a reduction in autophagic flux and gives rise to ER stress, the accumulation of dysfunctional mitochondria, oxidative stress, activation of AMPK, and a decrease in protein synthetic capacity. Moreover, these mice exhibit spontaneous activation of regenerative mechanisms that initiate acinar-to-ductal metaplasia (Chen et al. 2020). Cerulein induces mitophagy with the formation of autophagosomes in acinar cells; however, autophagosome-lysosome fusion is impaired due to altered levels of lysosomal-associated membrane protein 1 (LAMP-1), AMPK, and unc-51-like kinase (ULK-1), leading to autophagosome accumulation (incomplete autophagy). Lysosomal dysfunction plays a crucial role in AP, with abnormal processing and activation of histone and major lysosomal hydrolases and reduced levels of key lysosomal membrane proteins. LAMP-2 deficiency results in inflammation and acinar cell necrosis. Mitochondrial and lysosomal dysfunction act synergistically to promote impaired autophagy, thereby contributing to the development of AP Reduced levels of Rab9 and its membrane binding have been observed in rodent models of AP and human diseases. Overexpression of Rab9 in acinar cells stimulates non-classical autophagy and inhibits classical/LC3-mediated autophagy by upregulating ATG4B, a cysteine protease that cleaves LC3-II. Conversely, ATG5 deficiency leads to an increase in Rab9 in acinar cells. Inhibition of classical Rab9TG autophagy in the pancreas results in the accumulation of Rab9-positive vacuoles containing mitochondria, protein aggregates, and trans-Golgi markers. This transition to a non-classical pathway exacerbates experimental AP, causing damage to acinar cells. Rab9 regulates pancreatic autophagy, and there exists an antagonistic relationship between classical/LC3-mediated and non-classical/RAB9-mediated autophagy pathways in AP. Non-classical autophagy is unable to substitute for classical to prevent AP. Hence, the reduction of Rab9 in experimental and human AP is a protective response aimed at maintaining classical autophagy and reducing disease severity (Mareninova et al. 2022).

Whether mitophagy defects alone can drive the development of AP or if they merely enhance the susceptibility to AP is an important consideration in the pathophysiological context of this disease. Some evidence suggests that mitophagy defects could directly contribute to the onset of AP. Impaired mitophagy may lead to mitochondrial dysfunction, excessive ROS production, and subsequent induction of pancreatic cell death. In experimental models with specific mitophagy defects, there is an increased susceptibility to AP, indicating that mitophagy might be a crucial factor in the disease process (Zhang et al. 2021). However, these findings are primarily correlative, and a causal relationship remains to be firmly established. On the other hand, it is plausible that mitophagy defects do not directly cause AP but rather increase the pancreas’ susceptibility to injury (Pandol and Gottlieb 2022; Chen et al. 2023). In this scenario, mitophagy would be one of several factors that contribute to the overall risk of developing AP. This is in line with the multifactorial nature of AP, where genetic predisposition, environmental factors, and lifestyle choices all play a role (Mayerle et al. 2019). Defective mitophagy could exacerbate the injury response in the presence of other triggers, resulting in a more severe or frequent occurrence of AP. Mitophagy is likely to be a component of a multifactorial process. While mitophagy defects may not be the sole cause of AP, they could significantly modulate the disease’s progression and severity. This is supported by clinical observations where AP often occurs in the context of multiple risk factors. Moreover, therapeutic strategies targeting mitophagy could potentially mitigate the severity of AP, further suggesting a modifying rather than a causative role.

### The mitophagy-related targets and drugs for improving AP

Understanding the molecular mechanisms underlying the restoration of mitochondrial function, including mitochondrial dynamics and mitophagy, is of crucial significance for developing new therapeutic strategies for AP. We have compiled a summary of mitophagy-related targets and drugs for ameliorating AP in Table 2. Although the therapeutic efficacy of the mitophagy-related targets and drugs in AP has been verified through animal and cellular studies, and their underlying mechanisms have been elucidated, there currently exists a lacuna in their direct correlation with clinical AP and endeavors to translate these findings into clinical treatments. Nevertheless, there is a promising outlook that, in the future, these drug targets might indeed be applied to the clinical management of AP, potentially bringing about significant advancements in the treatment of this condition.

**Table 2:** The mitophagy-related targets and drugs for improving AP

Reagents	Model	Key mechanisms	Protective effects	References
Phosphate	Caerulein	Reducing Provoked Elevations in Intracellular Calcium	Preserving mitochondrial function	Farooq A, Hernandez L, Swain SM et al., 2022
Docosahexaenoic acid	Caerulein	Suppressing NADPH oxidase activity, reducing ROS level	Preventing mitochondrial dysfunction, inhibiting activation of necroptosis-regulating proteins	Jeong YK, Lee S, Lim JW, et al., 2017
Lycopene	Caerulein	Inhibiting NADPH oxidase activity, reducing ROS level	Preventing mitochondrial dysfunction and impaired autophagy, activating AMPK-dependent autophagy	Choi S, Kim H. 2020
N-acetylcysteine	Menadione	Inhibiting Oxidant-induced bioenergetic changes	Inhibiting the stimulation of basal respiration and oxidant-induced decrease of ATP turnover and spare respiratory capacity	Armstrong JA, Cash NJ, Ouyang Y, et al., 2018
N-methyl-4-isoleucine cyclosporin	Caerulein, EtOH + FA, taurocholic acid	Protecting mitochondrial function preserves bicarbonate transport mechanisms	Reducing serum amylase activity, pancreatic edema, necrosis, and leukocyte infiltration	Toth E, Maleth J, Zavogyan N et al., 2019
Simvastatin	Caerulein administration	Preserving autophagosome-lysosome fusion	Reducing inflammation and cell death, increasing stress-resistant mitochondrial population	Piplani H, Marek-Iannucci S, Sin J et al., 2019
Trehalose	L-arginine, caerulein, bile acid, AP-inducing diet	Stimulating autophagic flux and clearance of autophagic vacuoles	Preventing pancreatic necrosis and trypsinogen activation	Biczo G, Vegh ET, Shalbueva N et al., 2018

Mitochondria-related oxidative stress plays a significant role in mediating inflammatory signaling and cytokine expression in pancreatic acinar cells. It further leads to mitochondrial dysfunction and dysregulated autophagy, suggesting that oxidative stress might contribute to autophagic impairment in AP. Additionally, oxidative stress and acinar cell necroptosis are implicated in the pathology of severe AP. Some antioxidants have been found to protect against AP by alleviating mitochondrial dysfunction. For example, docosahexaenoic acid, a potent antioxidant with numerous conjugated double bonds that endows it with strong antioxidant properties, has been shown to inhibit EtOH/POA-induced necroptosis by suppressing NADPH oxidase activity, reducing ROS levels, preventing mitochondrial dysfunction, and inhibiting the activation of necroptosis-regulating proteins in AR42J cells (Ku et al. 2020). Lycopene, a bright-red carotenoid possessing potent antioxidant capabilities due to its high number of conjugated double bonds, has been found to inhibit EtOH/POA-induced mitochondrial dysfunction, zymogen activation, and IL-6 expression by suppressing NADPH oxidase-mediated ROS production in pancreatic acinar cells (Choi and Kim 2020; Lee et al. 2021). Moreover, oxidative stress has been linked to alterations in mitochondrial bioenergetics and modifications in pancreatic acinar cell death, resulting in a shift from apoptosis to necrosis. This shift appears to be associated with decreased mitochondrial spare respiratory capacity and ATP production, independent of CypD-sensitive MPTP formation (Armstrong et al. 2018).

Several other potential mitophagy-associated therapeutic targets for AP have been identified in recent studies. Phosphate supplementation has been found to enhance mitochondrial function and protect against experimental AP (Farooq et al. 2022). Downregulation of VMP1 has been demonstrated to alleviate mitochondrial degradation, indicating that VMP1 expression is crucial for mitophagy during AP. Dan Du et al. isolated compound 6 from the rhizomes of Dioscorea zingiberensis and demonstrated its potential as a candidate for alleviating mitochondrial dysfunction to prevent pancreatic necrosis (Du et al. 2017). E. Toth and J. Maleth et al. found that the novel cyclosporin A derivative N-methyl-4-isoleucine cyclosporin safeguards mitochondrial function in acinar and ductal cells, preserving the bicarbonate transport mechanisms in pancreatic ductal cells (Tóth et al. 2019). Simvastatin has been shown to boost autophagic flux to prevent pancreatic cell injury and AP by upregulating LAMP-1 and activating AMPK, which phosphorylates ULK-1, thereby increasing the formation of functional autolysosomes (Piplani et al. 2019). Administration of trehalose has also been found to largely prevent trypsinogen activation, necrosis, and other parameters of pancreatic injury in mice with L-arginine-induced AP.

## Conclusion and future perspective

The pathogenesis of AP is intricate, and a comprehensive understanding of the specific mechanisms involved is of utmost significance for the development of efficacious therapies. Currently, our knowledge of AP is confined to established molecules and genes, necessitating the exploration of novel molecules and targets to enhance our understanding of AP. This review centers on the pivotal role of mitophagy in the progression of AP, as it is regarded as the principal pathological mechanism of AP. By achieving a deeper comprehension of the mechanisms underlying mitophagy, we might be able to make breakthroughs in the study of AP. This review offers a systematic and comprehensive overview of the key pathobiological processes of the mitophagy pathway and the current research status of mitophagy in AP. This information could potentially contribute to the development of novel therapeutic strategies for AP. It is crucial to note that the relationship between mitophagy defects and AP is likely to be bidirectional. Initial mitophagy defects might contribute to the onset of pancreatitis, and the disease itself could further exacerbate mitophagy dysfunction through ongoing inflammatory processes and cellular stress. Defective mitophagy could also contribute to the development of AP. If mitochondria are not properly cleared, they could release cytochrome c and other pro-apoptotic factors, leading to cell death and inflammation, which are characteristic features of AP. On the other hand, mitophagy defects could also be a consequence of AP. The stress and inflammation associated with pancreatitis might overwhelm the cellular machinery responsible for mitophagy, resulting in its dysfunction. Nonetheless, further clinical trials are required to determine whether targeting mitochondrial autophagy can be utilized as an effective treatment for AP.

## Data Availability

No datasets were generated or analysed during the current study.

## Abbreviations

AP: Acute pancreatitis
ATG7: Autophagy-related protein 7
CAE: Caerulein
CerS: Ceramide synthases
CypD: Cyclophilin D
DAMPs: Danger-associated molecular patterns
DRP1: Dynamin-related protein 1
ER: Endoplasmic reticulum
EtOH/POA: Ethanol and palmitoleic acid
FKBP8 FK506: Binding Protein 8
FUNDC1 FUN14: Domain containing 1
HIF-1α: Hypoxia-inducible factor-1 alpha
LAMP-1: Lysosomal-associated membrane protein 1
LIR: LC3-interacting region
MIM: Mitochondrial inner membrane
MLKL: Mixed lineage kinase domain-like
MPTP: Membrane transition pore
NIX: NIP3-like protein X
NLRP3: NOD-like receptor pyrin domain-containing protein 3
OMM: Outer mitochondrial membrane
OPA-1: Optic Atrophy 1
PARK: Parkin
PINK1: Putative kinase protein 1
RIPK1: Receptor-interacting serine/threonine-protein kinase 3
ROS: Reactive oxygen species
SIRT: Sirtuins
TM: Terminal transmembrane
TOMM20: Translocase of the outer mitochondrial membrane complex subunit 20
TRPV4: Transient receptor potential vanilloid subfamily 4
ULK-1: Unc-51-like kinase
VMP1: Vacuole membrane protein-1
